# Quantitative T2 mapping of white matter: applications for ageing and cognitive decline

**DOI:** 10.1088/0031-9155/61/15/5587

**Published:** 2016-07-06

**Authors:** Michael J Knight, Bryony McCann, Demitra Tsivos, Serena Dillon, Elizabeth Coulthard, Risto A Kauppinen

**Affiliations:** 1 School of Experimental Psychology, 12a Priory Road, University of Bristol, Bristol, BS8 1TU, UK; 2 ReMemBr group, Institute for Clinical Neurosciences, University of Bristol, Level 1 Learning and Research Building, BS10 5NB, UK; 3 North Bristol NHS trust, Southmead Road, Westbury-on-Trym, Bristol, BS10 5NB, UK; 4 Clinical Research and Imaging Centre, University of Bristol, 60 St Michael’s Hill, Bristol, BS2 8DX, UK; mk13005@bristol.ac.uk

**Keywords:** T2 relaxation, MRI, brain, white matter, ageing, cognitive decline

## Abstract

In MRI, the coherence lifetime T2 is sensitive to the magnetic environment imposed by tissue microstructure and biochemistry *in vivo*. Here we explore the possibility that the use of T2 relaxometry may provide information complementary to that provided by diffusion tensor imaging (DTI) in ageing of healthy controls (HC), Alzheimer’s disease (AD) and mild cognitive impairment (MCI). T2 and diffusion MRI metrics were quantified in HC and patients with MCI and mild AD using multi-echo MRI and DTI. We used tract-based spatial statistics (TBSS) to evaluate quantitative MRI parameters in white matter (WM). A prolonged T2 in WM was associated with AD, and able to distinguish AD from MCI, and AD from HC. Shorter WM T2 was associated with better cognition and younger age in general. In no case was a reduction in T2 associated with poorer cognition. We also applied principal component analysis, showing that WM volume changes independently of  T2, MRI diffusion indices and cognitive performance indices. Our data add to the evidence that age-related and AD-related decline in cognition is in part attributable to WM tissue state, and much less to WM quantity. These observations suggest that WM is involved in AD pathology, and that T2 relaxometry is a potential imaging modality for detecting and characterising WM in cognitive decline and dementia.

## 1. Introduction

The detection of Alzheimer’s disease (AD) and stratification of dementia patients at the earliest possible stage is amongst the foremost current medical challenges. Typically, AD pathology is considered predominantly to affect grey matter (GM), in particular the limbic system (Braak and Braak 1991, Serrano-Pozo *et al* 2011), resulting in stress responses leading to inflammation (Halliday *et al* 2000, Blasko *et al* 2004, Tuppo and Arias 2005, Ricci *et al* 2012) and eventual atrophy (Frisoni *et al* 2010, Leung *et al* 2013, Tang *et al* 2014, Weiner *et al* 2015). Pro-inflammatory cytokines and cortisol levels are generally up-regulated in the brain in ageing and more so in cognitive decline, constituting a general state of stress for the afflicted tissue (Ricci *et al* 2012, Sudheimer *et al* 2014). Medial temporal lobe is often affected early, with accompanying loss of volume in the hippocampus and entorhinal cortices. However, medial temporal lobe atrophy is not an early indication of cognitive decline, as it occurs only after suffering stress or damage; atrophy is a relatively late event in the pathogenesis of AD and the pattern of atrophy is somewhat heterogeneous (Albert *et al* 2011, Jack *et al* 2011, McKhann *et al* 2011, Sperling *et al* 2011, Budson and Solomon 2012), with 11% of AD cases not involving the hippocampus at clinical presentation (Murray *et al* 2011, Whitwell *et al* 2012). The use of modalities more sensitive to white matter (WM) changes, and to stress or damage preceding tissue loss, may therefore be a powerful complement to those examining GM tissue loss. It is increasingly realized that AD, as well as cognitive decline and ageing in general, are associated with changes in WM at microstructural scale. Diffusion tensor imaging (DTI) has made significant contributions here (Fellgiebel and Yakushev 2011, Sexton *et al* 2011, Amlien and Fjell 2014). Diffusion kurtosis imaging, which extends DTI to consider the anisotropic kurtosis of diffusion, has also provided important data on the vulnerability of WM to age and AD-related changes (Fieremans *et al* 2013, Benitez *et al* 2014).

The diffusion tensor for water in a voxel reports on anisotropic barriers to diffusion on a length scale similar to that sampled by water molecules during the diffusion time in the pulse sequence. Often, increases in mean diffusivity (MD) or radial diffusivity (RD) are attributed to a degradation of the myelin sheath, whilst axial diffusivity (AxD) is considered an index of axonal damage. A similar argument applies to increases in fractional anisotropy (FA). Of course, many other structures impede the Brownian motion of water. Coming largely from tract-based spatial statistics (TBSS) analyses of DTI data, it has been consistently observed that MD, as well as RD and AxD are regionally increased relative to HC in both AD and MCI, whilst FA is generally reduced (Shu *et al* 2011, Palesi *et al* 2012, Hong *et al* 2013, Jacobs *et al* 2013, Santillo *et al* 2013, McMillan *et al* 2014). The increases in water diffusivity and decreases in FA, particularly when used in combination, have been able to distinguish amnestic MCI from AD in numerous studies (Wang *et al* 2012, Zhuang *et al* 2012, Liu *et al* 2013, Wang *et al* 2013). There is also evidence to suggest that WM alterations determinable by TBSS analysis of DTI data are more sensitive to amnestic MCI than global hippocampal volume measurements (Zhuang *et al* 2013, Remy *et al* 2015). However, the FA, despite its widespread use, has been reported as the diffusion index of least diagnostic power from TBSS analyses in AD and MCI (Clerx *et al* 2012, Nir *et al* 2013, Rowley *et al* 2013), and MD, AxD and RD are more sensitive to changes in AD and MCI. It therefore remains unclear precisely what nature of ‘WM changes’ are being observed in ageing and cognitive decline.

Recent studies have also revealed that WM changes in AD and cognitive decline may occur simultaneously with, or even before, GM tissue loss. The fornix and parahippocampal gyrus show signs of damage in AD independently of medial temporal lobe atrophy (Gold *et al* 2010, Salat *et al* 2010), whilst WM changes appear to be more widespread than GM tissue loss in AD (Agosta *et al* 2011) and subjective cognitive impairment (Selnes *et al* 2012). Taken together, these data are indicative that WM is under stress before GM volume losses becomes evident.

Alterations to WM tissue microstructure, that is, the degradation of supra-macromolecular structure large enough to impede Brownian translational motion, might also be expected to influence the coherence lifetime of nuclear spin phase, probed by T2. In MRI, the term T2 is commonly used to refer to the non-refocussable coherence lifetime, in distinction to chemistry, structural biology and physics, where T2 is reserved only for loss of spin phase coherence arising through stochastic modulation of the nuclear spin Hamiltonian (Abragam 1961, Luginbühl and Wüthrich 2002, Nicholas *et al* 2010). Terminology aside, T2 is heavily affected by ‘diffusion effects’, meaning diffusion of nuclear spins through magnetic field gradients created by the system’s own response to the static applied magnetic field (B0) of the MRI scanner (Kennan *et al* 1994, Boxerman *et al* 1995, Wharton and Bowtell 2012). Such field gradients are a result of materials within the system of different magnetic susceptibilities (Yablonskiy and Haacke 1994, Yablonskiy *et al* 1997). An example of particular relevance is the myelin sheath, which has a magnetic susceptibility different from aqueous solution (Argyridis *et al* 2013, Haacke *et al* 2015). Damage to, or breakdown of, such a supra-macromolecular structure alters the field lines it creates and thus alters T2. Interactions of water with macromolecules also influences T2 through exchange, such that alterations to tissue which alter water—macromolecule interactions may also alter T2. A net uptake of water may also increase T2, but with identifiable accompanying effects such as increased longitudinal relaxation time T1 and MR-observable proton density. It is known that WM changes with age (Kochunov *et al* 2012, Alves *et al* 2015), and various quantitative MRI methods in addition to DTI have been employed to study this. In particular, magnetization transfer (MT) and T1 show widespread age-related changes in WM (Draganski *et al* 2011, Callaghan *et al* 2014), as well as mean magnetic susceptibility (Haacke *et al* 2015). However, T2 has received less attention, despite its sensitivity to magnetic environment and ultimately to the physiology and biochemistry of the system under observation.

This study aimed to determine whether T2 may be useful in detecting differences between the WM of HCs, MCI and AD patients. We also used the same approaches to characterize distinct patterns of WM ageing in different tracts with T2 as a readout parameter.

## 2. Methods

### 2.1. Participants and cognitive tests

This study included 37 HC participants (22 female, mean age 67 years, age range from 49 to 87 years), 12 participants with MCI (4 female, mean age 74 years, age range 61–87 years) and 9 with mild AD (7 female, mean age 74 years, age range 56–91 years). MCI diagnosis was based on the criteria given by Petersen *et al* (1997). Participants also underwent the paired associative learning (PAL) task of the CANTAB toolbox (Soares *et al* 2014). This task requires that the participant recall the locations of various patterns, increasing in complexity as the task progresses, serving as a test of episodic memory. Cognitive testing was within 6 weeks of MRI. All participants gave informed consent and ethical approval was granted by the National Health Service Research Ethics Committee of North Bristol-Frenchay.

### 2.2. Image acquisition

All imaging was performed using a Siemens Magnetom Skyra 3T system equipped with a parallel transmit body coil and 32-channel head receiver array coil. The imaging protocol comprised a 3D T1-weighted MPRAGE, 2D multi-contrast spin-echo and 2D DTI with the following parameters: MPRAGE: coronal, TR 2200 ms, TE 2.42 ms, TI 900 ms, flip angle 9°, acquired resolution 0.68  ×  0.68  ×  1.60 mm^3^, acquired matrix size 152  ×  320  ×  144, reconstructed resolution 0.34  ×  0.34  ×  1.60 mm^3^ (after 2-fold interpolation in-plane by zero-filling in *k*-space), reconstructed matrix size 540  ×  640  ×  144, GRAPPA factor 2 (Griswold *et al* 2002), time 5:25. Multi-contrast spin-echo: TR 4500 ms, TE 12 ms, number of echoes 10, echo spacing 12 ms, acquired resolution 0.68  ×  0.68  ×  1.7 mm^3^ inclusive of 15% slice gap, acquired matrix size 152  ×  320, 34 slices, reconstructed resolution 0.34  ×  0.34  ×  1.7 mm^3^ (after 2-fold interpolation in-plane by zero-filling in *k*-space, and inclusive of 15% slice gap), reconstructed matrix size 540  ×  640, 34 slices, GRAPPA factor 2, time 11:07. DTI: axial, TR 3800 ms, TE 85.2 ms, Bval 1000 s mm^−2^, number of gradient directions 60 (full-sphere), acquired and reconstructed resolution 2  ×  2  ×  2 mm^3^ inclusive of 10% slice gap, matrix size 122  ×  122, 60 slices, GRAPPA factor 2, multi-band factor 2 (Feinberg *et al* 2010), time 4:30. Note that the T2 mapping did not have full-brain coverage, its anterior-posterior coverage only extending around 1 cm anterior and posterior to the head and tail of the hippocampus respectively, with the acquisition tilted such that the hippocampal body created an axis normal to the slice acquisition plane.

### 2.3. Image processing

Diffusion tensors were fitted using dtifit after eddy current correction using the eddy_correct program of fsl (Zhang *et al* 2001). T2 maps were computed by a voxel-wise fit of a mono-exponential function in a logarithmic space, excluding the first echo. This was done since the pulse sequence allows the passage of both spin and stimulated echoes due to the use of identical crusher gradients astride each refocusing pulse, though the first echo contains only spin echo contributions. Its exclusion therefore means that the time points to which the exponential function was fitted are the sum of spin and stimulated echoes. The exclusion of the first echo has previously been examined and applied by other authors (Maier *et al* 2003), who also demonstrated a marked improvement in quality of fit. This also has the effect of filtering out fast-decaying components of the signal. The final echo time of 120 ms meant that coherence in parenchyma decayed to ~25% of its initial value, a compromise between maintaining sufficient signal for a meaningful measurement but sampling enough of the decay for a precise T2 estimate. T1-weighted images were brain-extracted using the vbm8 toolbox (Ashburner 2012). Total WM volumes were calculated by segmentation of the T1-weighted MPRAGE scans in native space using fsl fast segmentation after brain-extraction by the vbm8 toolbox within spm.

### 2.4. TBSS analysis

To analyse the diffusion tensor and relaxometry data, the TBSS toolbox of fsl was used (Smith *et al* 2004, 2006). FA images were registered to the FMRIB58_FA standard template and the FA skeleton determined at a threshold of 0.2 after which permutation testing by the program randomize (Winkler *et al* 2014) was used to test for group differences. The demeaned age was included as a confound variable. 500 permutations were used. Group differences in FA, MD, RD and AxD were first tested for. Group differences in T2 were tested for by reciprocating the T2 maps to create R2 maps (T2  =  1/R2), performing 6-DOF registrations of the R2 maps to the T1-weighted images using the program flirt (Jenkinson and Smith 2001, Jenkinson *et al* 2002), then non-linearly registering the T1-weighted images to the native DTI space using fnirt (Smith *et al* 2004, Jenkinson *et al* 2012) (the reciprocal of the AxD image was used as a registration target) and applying the same transformations to the R2 maps. The R2 maps in native DTI space could then be fed into the TBSS analysis using the tbss_non_FA command and group differences assessed as before. R2 maps were chosen as registration targets due to their (broadly) similar contrast to a T1-weighted image. Likewise, the reciprocal of the L1 eigenvalue image has broadly similar contrast to a T1-weighted image and registration quality was excellent.

### 2.5. Statistical analysis

To test for differences in rates of WM ageing across tracts within the T2 FOV, the median T2 in each WM tract was calculated for each participant, using the Johns Hopkins University (JHU) WM tractography atlas (Wakana *et al* 2007, Hua *et al* 2008) at 95% probability as a primary mask, and superimposing the FA skeleton mask from the TBSS analysis as a secondary mask. Left and right hemispheric data were pooled. The effect of age and tract upon the T2 median in each tract and for each participant as defined above was then assessed by ANCOVA, as implemented in Matlab 2013b. The Bonferroni correction for multiple comparisons was also applied. The median T2 in each tract was used in preference to any other metric of ‘average’ since it is insensitive to outliers, and the T2 distribution in parenchyma generally deviates from normality. The median is the most robust and straightforward measure. The following 7 tracts were used in the analysis: Anterior thalamic radius (ATR), corticospinal tracts (CST), Inferior fronto-occipital fasciculus (IFOF), Inferior longitudinal fasciculus (ILoF), superior longitudinal fasciculus (SLoF), temporal part of superior longitudinal fasciculus (TSLoF) and forceps minor (FMi). These were the tracts in which differences between groups were detectable. The JHU atlas also contains the following tracts, which were not included in our analyses since the TBSS failed to detect any significant group differences: Cingulum (CG), hippocampal cingulum (CH), forceps major (FMa) and uncinate fasciculus (UF).

To examine the main sources of variances within the data, principal component analysis (PCA) was used, as implemented in Matlab 2013b. The median T2, FA, RD and AxD in the 7 WM tracts for which it was calculated were included, as well as age, total WM volume, and two measures of cognitive function, namely the total accuracy and mean reaction time from the PAL task of the CANTAB toolbox. Therefore, in total, 32 variables were included in the PCA. Variables were discarded if the magnitude of their PCA score vector in the space of the first four principal components was below the mean magnitude. Before analysis, variables were demeaned and normalized by their standard deviation.

## 3. Results

### 3.1. Regional T2 differences separating groups

**Figure 1.**
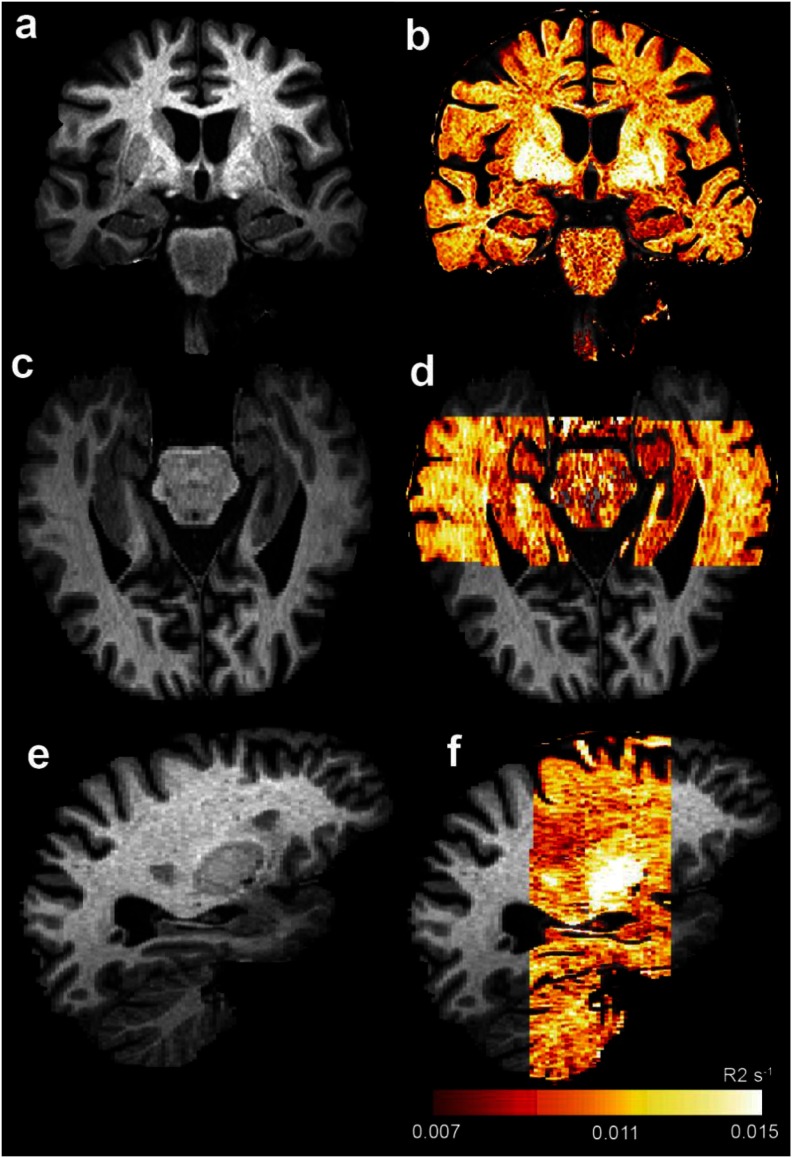
AD in the absence of hippocampal atrophy, but with hippocampal R2 reduction (T2 prolongation). Panels (a), (c) and (e) show coronal, axial and sagittal views of a T1-weighted MPRAGE scan. Panels (b), (d) and (f) show an R2 map (R2  =  1/ T2) overlaid onto this, demonstrating low R2 (high T2) in the hippocampus relative to cortical grey matter.

**Figure 2.**
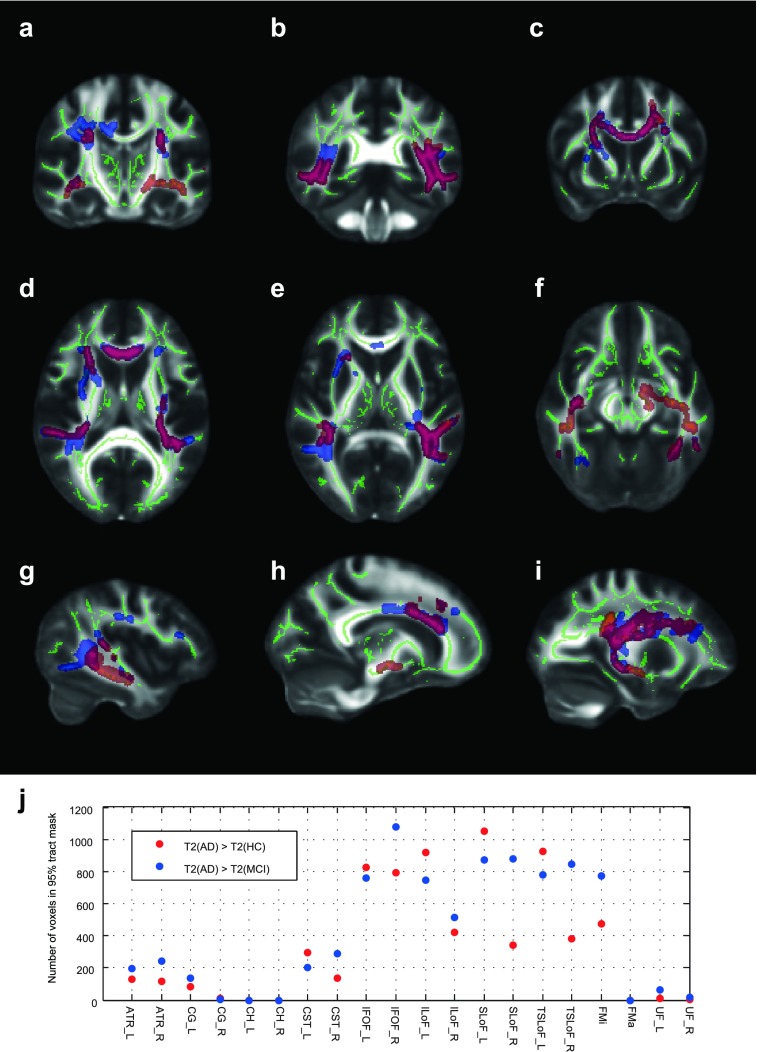
Changes in WM T2 identified by TBSS. Panels (a)–(i) show example slices demonstrating regions in which T2 could distinguish groups. The ‘FA skeleton’, identified from DTI data, is shown in green. Clusters identified by threshold-free cluster-enhancement (TFCE) confidently distinguishing groups at the 95% confidence level are shown with the following colours: red/yellow: T2(AD)  >  T2(NC); blue/light blue: T2(AD)  >  T2(MCI); purple/light green: T2(AD)  >  T2(HC) and T2(AD)  >  T2(MCI). Data are shown on the FMRIB58_FA standard FA template. Note that the FOV in T2 mapping was spatially limited to what is shown in figure 1. Panel (j) shows the number of voxels in all WM tracts of the JHU WM tractography atlas for which the TBSS analysis, using T2, could separate AD from HC or AD from MCI.

An example R2 (=1/T2) map is shown in figure 1, indicating the data quality and field of view analyzable. Using an FA skeletonisation threshold of 0.2 and a cutoff in cluster-corrected p-values of 0.05, the mono-exponential T2 maps were able to detect regional group differences, and these are shown in figure 2. In particular, we were able to localize regions in which the T2 for the AD group was longer than that of the MCI group, and for which the T2 of the AD group was longer than that of the NC group. In no case was the T2 of the NC group longer than that of the MCI or AD group, nor the MCI group T2 longer than that of the AD group; T2 only lengthened with diminishing cognitive function.

The main group differences in which T2 was increased in the AD group relative to both NC and MCI were in the FMi, IFOF, ILoF, SLoF and TSLoF. The ATR and CST were also affected, though to a lesser extent. The remaining tracts of the JHU WM tract atlas were negligibly affected, if at all. The number of voxels in the FA skeleton which distinguish groups in each WM tract is plotted in figure 2(j). The main group differences in which T2 was increased in the AD group relative to the NC group but not the MCI group were bilaterally in the temporal lobe (IFOF and ILoF), and uniquely in the left medial temporal lobe superior to the hippocampal head, in the ATR and CST. Many differences were not bilateral, or at least not symmetric. In general, T2 increases relative to controls were more pronounced, affecting larger areas, on the left hemisphere, which was particularly apparent in the ILoF, SLoF, TSLoF and CST. However, T2 increases separating the AD group from the MCI group were very much more symmetric, except in the ILoF. Some additional plots are provided in the supplementary information (stacks.iop.org/PMB/61/5587/mmedia). We did not detect group differences using DTI at the 95% significance level. Whilst slightly higher diffusivities and slightly lower FA were associated with AD and MCI, these were significant only at thresholds below 95% and are not presented in our main results.

### 3.2. The effect of age on T2 in different WM tracts

**Figure 3.**
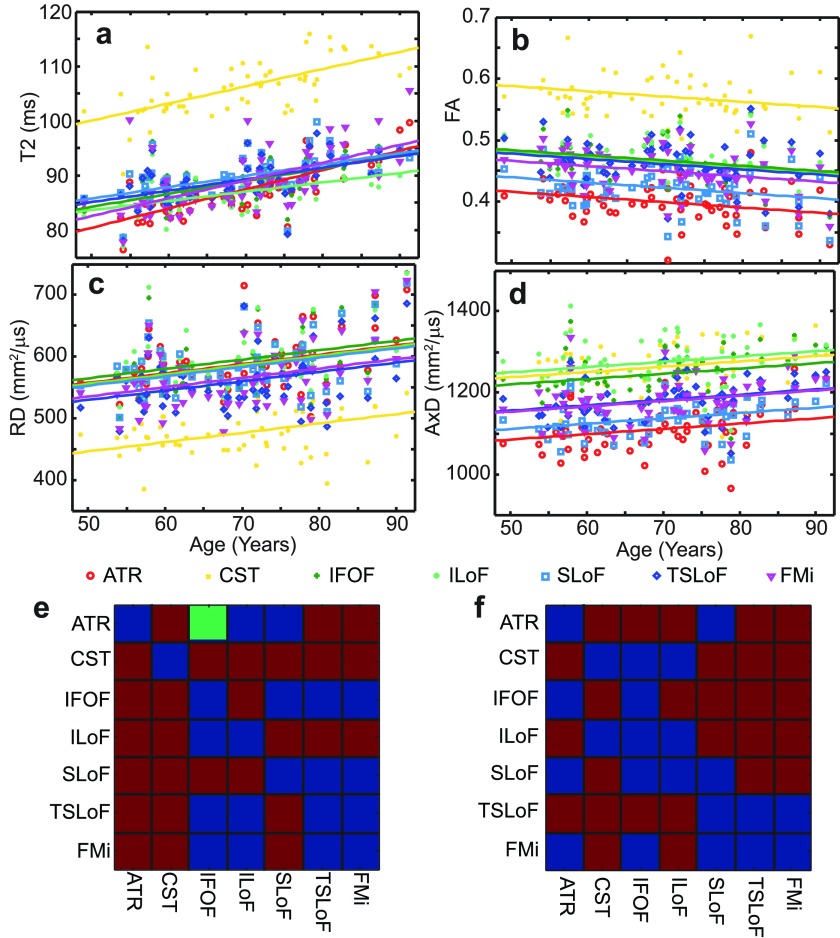
ANCOVA results for WM ageing rates across the entire cohort. The covariate was age. Panels (a)–(d) show the fits to T2, FA, RD and AxD as a function of age. For T2, separate fits were justified, whereas for FA, AxD and RD all gradients were indistinguishable so a common gradient was used. Panels (e) and (f) show the ‘separability’ matrices for the population marginal means and gradients. In panel (e), the lower left represents FA, the top right T2. In f, the lower left represents RD, the top right AxD. A red square denotes a pair of tracts distinguishable by population marginal mean at the 95% significance level. The green square denotes tracts separable by gradient with respect to age at the 95% significance level. Blue squares are for pairs of tracts indistinguishable at the 95% level.

By pooling the observations in each tract and calculating the tract-wise median for each imaging parameter (T2, FA, RD, AxD), we were able to delineate age effects in different WM tracts. Figure 3 shows ANCOVA results, comparing tract-wise T2, FA, RD and AxD medians within the entire cohort. Age was the covariate, and the 7 WM tracts analysed were the groups. We sought to determine whether the imaging parameters were different between different tracts, and whether they ‘aged’ at different rates.

The effect of age on T2 could be resolved at the 95% confidence level by both slope and intercept for each tract, whilst for DTI measures FA, RD and AxD we could only separate the intercepts at the 95% level. Therefore a common gradient was used for all tracts in those analyses. ANCOVA tables may be found in the supplementary information. T2 in all tracts increased with age, whilst FA always decreased. RD and AxD also increased with age. CST showed the longest T2 on average, (figure 3(a)), the highest FA (figure 3(b)) (by a smaller relative margin) and lowest RD (figure 3(c)) (again by a smaller relative margin than the T2 effect). However, the AxD were more similar in general. WM ageing rate (measured by the slope of the linear fit to tract-wise median T2 as a function of age in ANCOVA) was significantly higher than average in the ATR (*p*  =  0.021) and lower than average in the ILoF (*p*  =  0.026) (inclusive of Bonferroni correction). The ageing rates of the ILoF and ATR were also distinguishable at the 95% confidence level. Ageing rates are shown in figure 3(a).

### 3.3. Principle component analysis

**Figure 4.**
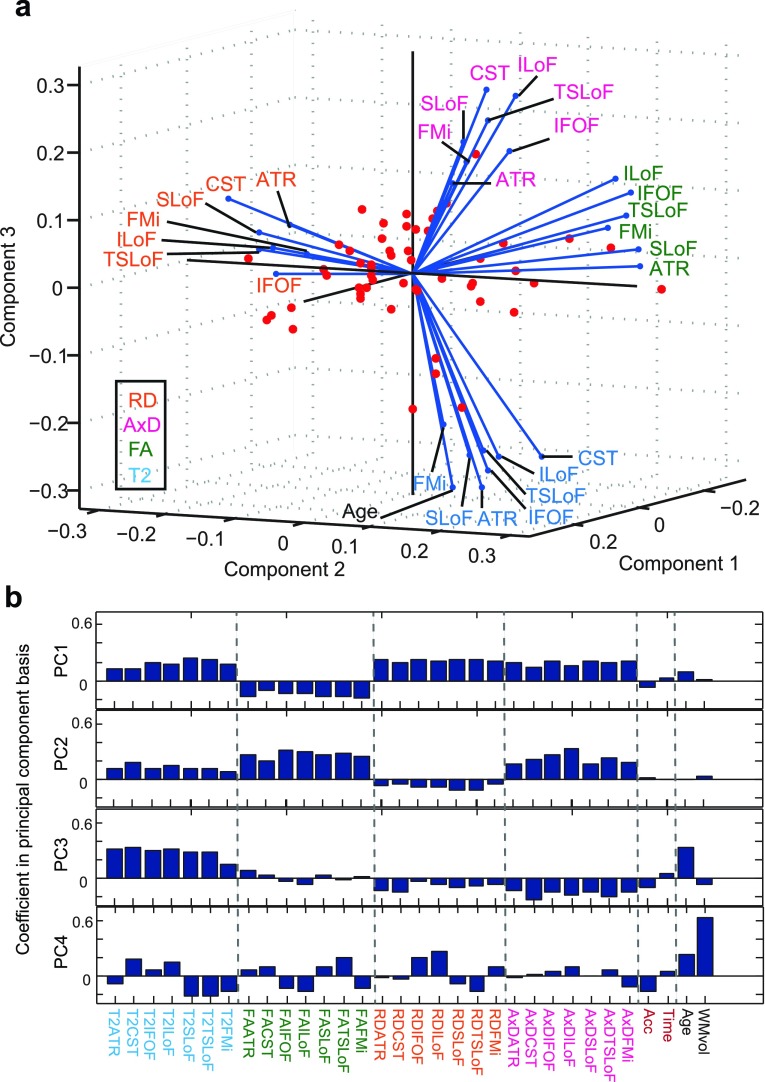
Principal component analysis of all data. Panel (a): the projections of the variables into the space of the first three PCs, showing which PCs are dominated by which variables. The variables are labelled by WM tract and coloured by variable type (see legend). Only if the projection in to the space of the first four PCs was large (see methods) are the variables displayed. Panel (b): contributions to the PC1-4 by all variables (PCA coefficients). The distinct types of variables are coloured as in panel (a) and separated by the dotted lines.

**Figure 5.**
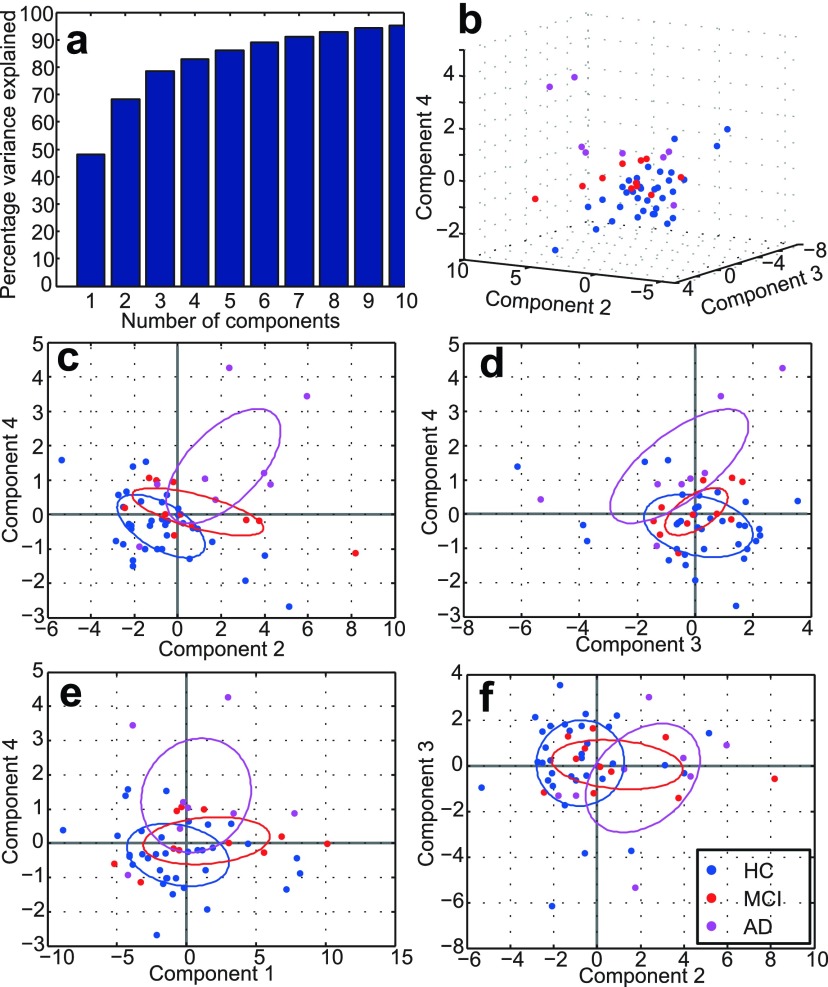
Resolving participant groups with four PCs. Panel (a) shows the cumulative amount of variance accounted for as a function of the number of PCs. Panels (b)–(f) show various scatter plots of the transformed data in the principal component basis. The axes show raw PCA values in the principal component basis. In (c)–(f), ellipses are drawn at the 1 standard deviation level.

The results of our PCA, using the median T2, FA, MD, RD and AxD in all 7 major WM tracts as well as age, WM volume, accuracy and reaction time in the PAL task are shown in figures 4 and 5. Only the variables making a substantial contribution (see methods section) to explaining the variance in the data are shown.

We found that 4 principal components (PC) were sufficient to explain 83% of the variance of the data (figure 5(a)), with rapidly diminishing returns thereafter. Figure 4(a) shows the contributions of different variables to the PC1–PC3 which are able to explain 78% variance. This figure shows that FA, RD and AxD make distinct contributions to explaining the variance of the data. T2 makes a similar contribution to age, which may be resolved in the PC4 (figure 4(b)). The distinct variable types (FA, RD, AxD, T2) are well resolved from one another in three PCs and the PAL variables, age and WM volume distinguished by the PC4. Although in the PC1, the diffusivity indices RD and AxD are similar, they are fully resolved in the PC2, demonstrating their distinct information content. T2 projects positively and roughly diagonally into the space of the PC1-3.

We also found that WM volume, as well the PAL performance indices, made large contributions mainly in higher PCs. The WM volume dominates the PC4, whilst age makes large contributions to the PC3 and PC4. PAL reaction time makes a small positive projection diagonally across the PC(1,3,4) space, but is largely disassociated from WM volume. Positive T2 coefficients in PC1 and PC3 are associated with negative coefficients for PAL accuracy and positive coefficients for PAL time. This means that longer T2 implies poorer pattern recognition and longer reaction times. This is of particular interest as the TBSS identified regional T2 increases to be associated with AD.

We also examined whether the unsupervised PCA, having reduced the 32-dimensional data to 4 dimensions, would reveal potential differences between participant groups. Figures 5(b)–(f) show scatter plots demonstrating that this is indeed the case, at least to some extent. Note that the three groups are not separated in PC1, but PC2-4 show better separation.

## 4. Discussion

We have found increases in the coherence lifetime of nuclear spin phase in WM, heuristically described by a mono-exponential decay time constant at long echo times (i.e. T2), to be associated with ageing and poorer performance in cognitive tasks, the increases being regionally greater in AD. We have also found that both the tract-wise median T2 and its rate of change with respect to age differs in different WM tracts.

Our data also indicate, consistent with the literature, that older age is associated with lower FA in all WM tracts examined in the sampled age range (Kochunov *et al* 2012), but with higher diffusivity measures (MD, AxD, RD) (Bennett *et al* 2010). The T2 effect size was larger, and also able to distinguish groups. To some extent, this may be attributable to both the signal-to-noise ratio and resolution in the experiments. We dedicate more time to our T2 experiment, yet it is required to fit fewer parameters. To obtain DTI data with comparable resolution to our T2 data whilst maintaining comparable precision for diffusion tensor parameters, a long experiment would be necessary (and challenging with echo-planar readout due to resolution requirements). To detect the changes seen with T2 using DTI instead, the latter technique would need substantially improved sensitivity, or otherwise larger sample sizes would be obliged.

### 4.1. Sensitivity of T2 to WM ageing and AD

Our TBSS analysis identified regions within WM in which T2 was longer in early AD patients, whose PAL accuracy and reaction times were significantly worse than MCI or HC. Our PCA analysis, using an atlas-based labelling of WM tracts, and not limited to the regions separating participant groups, confirmed that, independent of age or WM volume, high T2 in WM is associated with both poorer PAL accuracy and longer reaction times to identify patterns. This is interesting given recent literature indicating that widespread WM changes may occur concurrently with, or even before, GM tissue loss in cognitive decline and AD (Gold *et al* 2010, Salat *et al* 2010, Agosta *et al* 2011, Heise *et al* 2011, Selnes *et al* 2012). Magnetization transfer (MT) saturation has also been shown to correlate negatively with age extensively in WM (Draganski *et al* 2011, Callaghan *et al* 2014). The age-related MT saturation decreases reported in the latter studies were interpreted as being due to reduction in myelin content.

We postulate that the changes in T2 we have observed are also due to microstructural degradation of WM, by which the large induced field gradients which attenuate the signal by randomizing spin phase are lost. Equally then, we follow the existing literature that AD is as much a disease of WM as GM (Sexton *et al* 2011, Sachdev *et al* 2013), even if abnormality becomes evident at different times. Our PCA analysis was able to delineate the effect of WM volume loss from T2 increase (as well as from changes in diffusivity and FA). This evidences the postulate that WM structural changes, not WM loss, explain the data. However, with the current MRI approach we cannot separate cerebrovascular alterations in WM from changes in microstructural changes, such as change in myelination.

Generally, one can anticipate more rapid spin phase decoherence as being a consequence of field perturbers, as stated in the introduction. The myelin sheath, amongst other entities, is such a structure, whose different susceptibility relative to its surroundings has received substantial attention (Wharton and Bowtell 2012, Rudko *et al* 2014). A short T2 in WM may then likely to be indicative of healthy tissue, whose summative field lines conspire to dephase magnetization rather quickly. As such destruction of the ordered myelin sheath may account for our T2 observations.

Capillaries and small vessels tend to lie parallel to axons (Carmeliet and Tessier-Lavigne 2005, Guthrie 2007, Eichmann and Thomas 2013), so there is likely to be an orientation-dependent dephasing related to cerebrovascular state and architecture in WM. Alteration of cerebrovascular architecture, which is known to occur which age and differently in AD and cognitive impairment in general (Gorelick *et al* 2011, Honjo *et al* 2012), may be reflected in the increased WM T2 observed here. However, the effect may be small, considering blood volume. It is also possible that reduced cerebral perfusion may lengthen coherence lifetime since molecules in tissues may be less affected by field lines created by flowing paramagnetic deoxyhaemoglobin (Kennan *et al* 1994, Boxerman *et al* 1995) (diffusion-mediated non-refocussable dephasing is distinct from the BOLD effect though). Cerebral perfusion is reasonably stable in the age range sampled (Parkes *et al* 2004, Biagi *et al* 2007), though is affected by AD (Alsop *et al* 2010, Wolk and Detre 2012), being reduced generally in parietal areas. The reasonable stability of WM perfusion versus the strong correlation of T2 with age argues against this mechanism. The accumulation of paramagnetic substances in tissue, such as iron (e.g. in the form of ferritin), may also reduce T2 substantially (Mitsumori *et al* 2007). Conversely, a loss of tissue iron may account for lengthened T2, but there is scant evidence of iron loss with age as a general phenomenon. Rather, the opposite is generally reported (Qin *et al* 2011, Daugherty and Raz 2015), making non-heme iron an unlikely candidate to explain our observations.

Therefore our working hypothesis is that the T2 increases observed with age, and regional disproportionate increases in AD patients, are due to WM degradation involving breakdown of the ordered myelin sheath and/or alterations to microvascular structure.

### 4.2. Why should different tracts show different age-associated changes?

The observation of large age-related changes in T2 in the ATR and FMi, but smaller age-related changes in T2 in the ILoF in the age range sampled is evidence of different ageing rates in WM tracts, albeit derived from cross-sectional data. Equally, it is a fine demonstration of the power of quantitative T2 to detect changes which are likely to be microstructural in origin, such as Wallerian degeneration (Alves *et al* 2015). We interpret the finding as adding to the evidence that not all WM is the same. Different ageing rates may also affect information processing and a variety of memory processes.

The T2 was longest in the CST and shortest in the ATR, irrespective of age and cognitive state. In part, this is likely due to inherent differences in cell biology and biochemistry between the regions, such as g-ratios, axon diameters, water content etc (Wharton and Bowtell 2012). The CST and ATR are also distinguished by DTI, in particular the high FA of the CST and low AxD of the ATR. Myelinated axon diameters are heterogeneous within and across different WM tracts, with generally larger diameters in the SLoF and IFOF, but generally smaller diameters in the corpus callosum (including FMi) (Liewald *et al* 2014). The CST has a broad distribution of axonal diameters similar to the SLoF. However, the magnetic resonance physics are also likely to be involved. In particular, this observation is likely in part to be a consequence of coherence lifetime anisotropy, meaning that T2 (again, used interchangeably with coherence lifetime) is a function of the angle between the system under observation and the applied magnetic field (Knight *et al* 2015). This is the case as long as diffusion effects contribute to coherence lifetime, for they depend on magnetic susceptibility differences which create anisotropic field lines (Majumdar and Gore 1988, Borgia *et al* 1996, Hürlimann 1998, Sen and Axelrod 1999, Audoly *et al* 2003, Cho *et al* 2009). It is also the case in any motionally restricted systems.

Magnetic susceptibility is a tensor quantity (Haacke *et al* 2015), and as such depends on orientation with respect to the applied magnetic field. It can be measured by susceptibility tensor imaging (Liu 2010), and its effects have been studied extensively in gradient echo experiments. By similar reasoning, field lines at microscopic scale created by tensorial magnetic susceptibility also depend on system orientation with respect to B0, and as already alluded to make a substantial contribution to spin phase decoherence. When the long axis of a system’s susceptibility tensor is parallel to the B0, coherence lifetime is maximized (Yablonskiy and Haacke 1994). When perpendicular, it is minimized. The long axis of the susceptibility tensor, in WM, is to a good approximation parallel to the tract, as has been demonstrated (Liu *et al* 2012). The CST, with participants in the supine position, are *approximately* parallel to B0, whereas the ATR is *approximately* perpendicular to B0.

### 4.3. Drawbacks

This study has weakness both in terms of the size of clinical cohort and imaging techniques used. Firstly, the MCI and AD participant numbers were rather low, and the study was cross-sectional. Since characterizing effects of age across a reasonably broad age range was a study aim, a cross-sectional design is preferable, despite the confounds it introduces. However, we were able to observe extensive T2 effects, which were distinct from diffusion and age effects as shown by PCA. This is a promising sign that quantitative T2 is able to detect AD pathology in WM. The ability to detect the ‘spread’ or even existence of WM abnormality in AD by prolonged T2 may have future clinical utility. An advantage over DTI is the ease with which T2 measurements can be made with higher resolution, to which arguably some of our findings may be attributable. The drawbacks of TBSS apply (Bach *et al* 2014), for example that the FA skeleton is unlikely to represent the ‘tract centre’. Our cohort was not gender-balanced (22/37 HC female, 4/12 MCI female, 7/9 AD female). It is not impossible that this imbalance contributes to the group differences seen in T2 and/or the failure to observe significant group differences by DTI. However, gender differences seen by DTI, in normal ageing and AD, have generally been small and heterogeneous, if seen at all (Dunst *et al* 2014) (leaving us probably underpowered to detect them). Only in large studies have consistent effects been detectable (Ingalhalikar *et al* 2014). The use of T2 to examine gender differences is poorly represented in the literature, though T2 has been reported to be consistent between males and females (Siemonsen *et al* 2008). Atrophy patterns between males and females are almost identical, so there are grounds to suspect that the underlying parameters are also similar (Fjell *et al* 2009).

Secondly, we have heuristically described loss of spin phase coherence by a mono-exponential function, from the second (24 ms) echo onwards. There is literature supporting the view that decoherence in WM is better (albeit still heuristically) described by bi-exponential kinetics (MacKay *et al* 1994, Whittall *et al* 1997, Alonso-Ortiz *et al* 2015). There exists a correlation between the population of the short-T2 component and luxol fast blue optical density in formalin-fixed ex-vivo human brains (Laule *et al* 2008). Motivated by this, if a 2-state slow-exchange model be assumed, the time constants may be interpreted as belonging to ‘free water’ and ‘myelin-associated water’ (or the Eigenvalues of a magnetization evolution matrix outside the slow-exchange limit). This has been powerful in addressing and quantifying myelin sheath damage in multiple sclerosis (amongst other applications) (Alonso-Ortiz *et al* 2015). However, we have an inadequate number of TE data points to extract parameters with sufficient precision for such a model to be useful, in part due to the relatively high resolution we used, and in part the exclusion of the first (spin) echo due to identical crusher gradients throughout the experiment; fast-decaying components of the kinetics would be lost before we observe (or even refocus) them. In principle, the first echo could be retained if a more sophisticated fitting approach such as the extended phase graph method were used (Weigel 2015), but we would be including only a single extra data point which may therefore not markedly increase the information content. Our DTI data processing followed similar principles to the relaxation data modelling, treating each voxel as having a single ‘effective’ diffusion tensor, rather than multiple slowly or non-exchanging states of water in distinct ‘compartments’ with distinct diffusion tensors. This is entirely normal in DTI. Spin phase decoherence, as such whatever parameters in any model for it, are somewhat pulse-sequence and magnetic field dependent. DTI suffers this to a lesser extent. Any future clinical application would need to keep this in mind.

Thirdly, our field of view in our T2 experiment only covered part of the brain, missing some of the occipital and frontal lobes (but including most regions in which AD pathology is known). Again, coverage was sacrificed for resolution. Continued improvements in MRI technology and pulse sequences may in future ameliorate this. Most major WM tracts are partially or fully covered, as well as the most of the temporal lobe and entire limbic system, cerebellum and brain stem.

## 5. Conclusions

In all, it appears that T2 in WM is affected by age in later life to a similar or greater extent than common DTI parameters and may be a powerful complement to other more popular imaging techniques. WM is an appealing source of imaging biomarkers since damage and stress alter its magnetic environment in ways that are not so in GM. For this reason, continued deployment of MRI techniques which detect changes in magnetic environment in response to stress of damage, rather than volume loss occurring afterwards, is likely to be powerful in detecting and characterizing various pathologies.

## References

[pmbaa2ad9bib001] Abragam A (1961). Principles of Nuclear Magnetism.

[pmbaa2ad9bib002] Agosta F, Pievani M, Sala S, Geroldi C, Galluzzi S, Frisoni G B, Filippi M (2011). White matter damage in Alzheimer disease and its relationship to gray matter atrophy. Radiology.

[pmbaa2ad9bib003] Albert M S (2011). The diagnosis of mild cognitive impairment due to Alzheimer’s disease: recommendations from the National Institute on Aging-Alzheimer’s Association workgroups on diagnostic guidelines for Alzheimer’s disease. Alzheimers Dement..

[pmbaa2ad9bib004] Alonso-Ortiz E, Levesque I R, Pike G B (2015). MRI-based myelin water imaging: a technical review. Magn. Reson. Med..

[pmbaa2ad9bib005] Alsop D C, Dai W, Grossman M, Detre J A (2010). Arterial spin labeling blood flow MRI: its role in the early characterization of Alzheimer’s disease. J. Alzheimers Dis..

[pmbaa2ad9bib006] Alves G S, Oertel Knochel V, Knochel C, Carvalho A F, Pantel J, Engelhardt E, Laks J (2015). Integrating retrogenesis theory to Alzheimer’s disease pathology: insight from DTI-TBSS investigation of the white matter microstructural integrity. Biomed. Res. Int..

[pmbaa2ad9bib007] Amlien I K, Fjell A M (2014). Diffusion tensor imaging of white matter degeneration in Alzheimer’s disease and mild cognitive impairment. Neuroscience.

[pmbaa2ad9bib008] Argyridis I, Li W, Johnson G A, Liu C (2013). Quantitative magnetic susceptibility of the developing mouse brain reveals microstructural changes in the white matter. Neuroimage.

[pmbaa2ad9bib009] Ashburner J (2012). SPM: a history. Neuroimage.

[pmbaa2ad9bib010] Audoly B, Sen P N, Ryu S, Song Y Q (2003). Correlation functions for inhomogeneous magnetic field in random media with application to a dense random pack of spheres. J. Magn. Reson..

[pmbaa2ad9bib011] Bach M, Laun F B, Leemans A, Tax C M, Biessels G J, Stieltjes B, Maier-Hein K H (2014). Methodological considerations on tract-based spatial statistics (TBSS). Neuroimage.

[pmbaa2ad9bib012] Benitez A, Fieremans E, Jensen J H, Falangola M F, Tabesh A, Ferris S H, Helpern J A (2014). White matter tract integrity metrics reflect the vulnerability of late-myelinating tracts in Alzheimer’s disease. Neuroimage Clin..

[pmbaa2ad9bib013] Bennett I J, Madden D J, Vaidya C J, Howard D V, Howard J H (2010). Age-related differences in multiple measures of white matter integrity: a diffusion tensor imaging study of healthy aging. Hum. Brain Mapp..

[pmbaa2ad9bib014] Biagi L, Abbruzzese A, Bianchi M C, Alsop D C, Del Guerra A, Tosetti M (2007). Age dependence of cerebral perfusion assessed by magnetic resonance continuous arterial spin labeling. J. Magn. Reson. Imaging.

[pmbaa2ad9bib015] Blasko I, Stampfer-Kountchev M, Robatscher P, Veerhuis R, Eikelenboom P, Grubeck-Loebenstein B (2004). How chronic inflammation can affect the brain and support the development of Alzheimer’s disease in old age: the role of microglia and astrocytes. Aging Cell.

[pmbaa2ad9bib016] Borgia G C, Brown R J S, Fantazzini P (1996). The effect of diffusion and susceptibility differences on T2 measurements for fluids in porous media and biological tissues. Magn. Reson. Imaging.

[pmbaa2ad9bib017] Boxerman J L, Hamberg L M, Rosen B R, Weisskoff R M (1995). MR contrast due to intravascular magnetic susceptibility perturbations. Magn. Reson. Med..

[pmbaa2ad9bib018] Braak H, Braak E (1991). Neuropathological stageing of Alzheimer’s related-changes. Acta Neuropathol..

[pmbaa2ad9bib019] Budson A E, Solomon P R (2012). New diagnostic criteria for Alzheimer’s disease and mild cognitive impairment for the practical neurologist. Pract. Neurol..

[pmbaa2ad9bib020] Callaghan M F (2014). Widespread age-related differences in the human brain microstructure revealed by quantitative magnetic resonance imaging. Neurobiol. Aging.

[pmbaa2ad9bib021] Carmeliet P, Tessier-Lavigne M (2005). Common mechanisms of nerve and blood vessel wiring. Nature.

[pmbaa2ad9bib022] Cho H, Ryu S, Ackerman J L, Song Y Q (2009). Visualization of inhomogeneous local magnetic field gradient due to susceptibility contrast. J. Magn. Reson..

[pmbaa2ad9bib023] Clerx L, Visser P J, Verhey F, Aalten P (2012). New MRI markers for Alzheimer’s disease: a meta-analysis of diffusion tensor imaging and a comparison with medial temporal lobe measurements. J. Alzheimers Dis..

[pmbaa2ad9bib024] Daugherty A M, Raz N (2015). Appraising the role of iron in brain aging and cognition: promises and limitations of MRI methods. Neuropsychol. Rev..

[pmbaa2ad9bib025] Draganski B, Ashburner J, Hutton C, Kherif F, Frackowiak R S, Helms G, Weiskopf N (2011). Regional specificity of MRI contrast parameter changes in normal ageing revealed by voxel-based quantification (VBQ). Neuroimage.

[pmbaa2ad9bib026] Dunst B, Benedek M, Koschutnig K, Jauk E, Neubauer A C (2014). Sex differences in the IQ-white matter microstructure relationship: a DTI study. Brain Cogn..

[pmbaa2ad9bib027] Eichmann A, Thomas J L (2013). Molecular parallels between neural and vascular development. Cold Spring Harb. Perspect. Med..

[pmbaa2ad9bib028] Feinberg D A (2010). Multiplexed echo planar imaging for sub-second whole brain FMRI and fast diffusion imaging. PLoS One.

[pmbaa2ad9bib029] Fellgiebel A, Yakushev I (2011). Diffusion tensor imaging of the hippocampus in MCI and early Alzheimer’s disease. J. Alzheimers Dis..

[pmbaa2ad9bib030] Fieremans E (2013). Novel white matter tract integrity metrics sensitive to Alzheimer disease progression. AJNR Am. J. Neuroradiol..

[pmbaa2ad9bib031] Fjell A M (2009). Minute effects of sex on the aging brain: a multisample magnetic resonance imaging study of healthy aging and Alzheimer’s disease. J. Neurosci..

[pmbaa2ad9bib032] Frisoni G B, Fox N C, Jack C R, Scheltens P, Thompson P M (2010). The clinical use of structural MRI in Alzheimer disease. Nat. Rev. Neurol..

[pmbaa2ad9bib033] Gold B T, Powell D K, Andersen A H, Smith C D (2010). Alterations in multiple measures of white matter integrity in normal women at high risk for Alzheimer’s disease. Neuroimage.

[pmbaa2ad9bib034] Gorelick P B (2011). Vascular contributions to cognitive impairment and dementia: a statement for healthcare professionals from the American heart association/American stroke association. Stroke.

[pmbaa2ad9bib035] Griswold M A, Jakob P M, Heidemann R M, Nittka M, Jellus V, Wang J M, Kiefer B, Haase A (2002). Generalized autocalibrating partially parallel acquisitions (GRAPPA). Magn. Reson. Med..

[pmbaa2ad9bib036] Guthrie S (2007). Patterning and axon guidance of cranial motor neurons. Nat. Rev. Neurosci..

[pmbaa2ad9bib037] Haacke E M, Liu S, Buch S, Zheng W, Wu D, Ye Y (2015). Quantitative susceptibility mapping: current status and future directions. Magn. Reson. Imaging.

[pmbaa2ad9bib038] Halliday G, Robinson S R, Shepherd C, Kril J (2000). Alzheimer’s disease and inflammation: a review of cellular and therapeutic mechanisms. Clin. Exp. Pharmacol. Physiol..

[pmbaa2ad9bib039] Heise V, Filippini N, Ebmeier K P, Mackay C E (2011). The APOE varepsilon4 allele modulates brain white matter integrity in healthy adults. Mol. Psychiatry.

[pmbaa2ad9bib040] Hong Y J, Yoon B, Lim S C, Shim Y S, Kim J Y, Ahn K J, Han I W, Yang D W (2013). Microstructural changes in the hippocampus and posterior cingulate in mild cognitive impairment and Alzheimer’s disease: a diffusion tensor imaging study. Neurol. Sci..

[pmbaa2ad9bib041] Honjo K, Black S E, Verhoeff N P (2012). Alzheimer’s disease, cerebrovascular disease, and the beta-amyloid cascade. Can. J. Neurol. Sci..

[pmbaa2ad9bib042] Hua K (2008). Tract probability maps in stereotaxic spaces: analyses of white matter anatomy and tract-specific quantification. Neuroimage.

[pmbaa2ad9bib043] Hürlimann M D (1998). Effective gradients in porous media due to susceptibility differences. J. Magn. Reson..

[pmbaa2ad9bib044] Ingalhalikar M (2014). Sex differences in the structural connectome of the human brain. Proc. Natl Acad. Sci. USA.

[pmbaa2ad9bib045] Jack C R, Albert M S, Knopman D S, McKhann G M, Sperling R A, Carrillo M C, Thies B, Phelps C H (2011). Introduction to the recommendations from the National Institute on Aging-Alzheimer’s association workgroups on diagnostic guidelines for Alzheimer’s disease. Alzheimers Dement..

[pmbaa2ad9bib046] Jacobs H I, van Boxtel M P, Gronenschild E H, Uylings H B, Jolles J, Verhey F R (2013). Decreased gray matter diffusivity: a potential early Alzheimer’s disease biomarker?. Alzheimers Dement..

[pmbaa2ad9bib047] Jenkinson M, Bannister P, Brady M, Smith S (2002). Improved optimization for the robust and accurate linear registration and motion correction of brain images. Neuroimage.

[pmbaa2ad9bib048] Jenkinson M, Beckmann C F, Behrens T E, Woolrich M W, Smith S M (2012). Fsl. Neuroimage.

[pmbaa2ad9bib049] Jenkinson M, Smith S (2001). A global optimisation method for robust affine registration of brain images. Med. Image Anal..

[pmbaa2ad9bib050] Kennan R P, Zhong J, Gore J C (1994). Intravascular susceptibility contrast mechanisms in tissues. Magn. Reson. Med..

[pmbaa2ad9bib051] Knight M J, Wood B, Coulthard E, Kauppinen R A (2015). Anisotropy of spin-echo T2 relaxation by magnetic resonance imaging in the human brain. in vivo Biomed. Spectrosc. Imag..

[pmbaa2ad9bib052] Kochunov P, Williamson D E, Lancaster J, Fox P, Cornell J, Blangero J, Glahn D C (2012). Fractional anisotropy of water diffusion in cerebral white matter across the lifespan. Neurobiol. Aging.

[pmbaa2ad9bib053] Laule C, Kozlowski P, Leung E, Li D K B, MacKay A L, Moore G R W (2008). Myelin water imaging of multiple sclerosis at 7°T: correlations with histopathology,. Neuroimage.

[pmbaa2ad9bib054] Leung K K, Bartlett J W, Barnes J, Manning E N, Ourselin S, Fox N C (2013). Cerebral atrophy in mild cognitive impairment and Alzheimer disease: rates and acceleration. Neurology.

[pmbaa2ad9bib055] Liewald D, Miller R, Logothetis N, Wagner H J, Schuz A (2014). Distribution of axon diameters in cortical white matter: an electron-microscopic study on three human brains and a macaque. Biol. Cybern..

[pmbaa2ad9bib056] Liu C L (2010). Susceptibility Tensor Imaging. Magn. Reson. Med..

[pmbaa2ad9bib057] Liu C, Li W, Wu B, Jiang Y, Johnson G A (2012). 3D fiber tractography with susceptibility tensor imaging. Neuroimage.

[pmbaa2ad9bib058] Liu J, Yin C, Xia S, Jia L, Guo Y, Zhao Z, Li X, Han Y, Jia J (2013). White matter changes in patients with amnestic mild cognitive impairment detected by diffusion tensor imaging. PLoS One.

[pmbaa2ad9bib059] Luginbühl P, Wüthrich K (2002). Semi-classical nuclear spin relaxation theory revisited for use with biological macromolecules. Prog. Nucl Magn. Reson. Spectrosc..

[pmbaa2ad9bib060] MacKay A, Whittall K, Adler J, Li D, Paty D, Graeb D (1994). *In vivo* visualization of myelin water in brain by magnetic resonance. Magn. Reson. Med..

[pmbaa2ad9bib061] Maier C F, Tan S G, Hariharan H, Potter H G (2003). T2 quantitation of articular cartilage at 1.5 T. J. Magn. Reson. Imaging.

[pmbaa2ad9bib062] Majumdar S, Gore J C (1988). Studies of diffusion in random fields produced by variations in susceptibility. J. Magn. Reson..

[pmbaa2ad9bib063] McKhann G M (2011). The diagnosis of dementia due to Alzheimer’s disease: recommendations from the National Institute on Aging-Alzheimer’s Association workgroups on diagnostic guidelines for Alzheimer’s disease. Alzheimers Dement..

[pmbaa2ad9bib064] McMillan C T, Avants B B, Cook P, Ungar L, Trojanowski J Q, Grossman M (2014). The power of neuroimaging biomarkers for screening frontotemporal dementia. Hum. Brain Mapp..

[pmbaa2ad9bib065] Mitsumori F, Watanabe H, Takaya N, Garwood M (2007). Apparent transverse relaxation rate in human brain varies linearly with tissue iron concentration at 4.7 T. Magn. Reson. Med..

[pmbaa2ad9bib066] Murray M E, Graff-Radford N R, Ross O A, Petersen R C, Duara R, Dickson D W (2011). Neuropathologically defined subtypes of Alzheimer’s disease with distinct clinical characteristics: a retrospective study. Lancet Neurol..

[pmbaa2ad9bib067] Nicholas M P, Eryilmaz E, Ferrage F, Cowburn D, Ghose R (2010). Nuclear spin relaxation in isotropic and anisotropic media. Prog. Nucl Magn. Reson. Spectrosc..

[pmbaa2ad9bib068] Nir T M, Jahanshad N, Villalon-Reina J E, Toga A W, Jack C R, Weiner M W, Thompson P M (2013). Effectiveness of regional DTI measures in distinguishing Alzheimer’s disease, MCI, and normal aging. Neuroimage Clin..

[pmbaa2ad9bib069] Palesi F (2012). DTI and MR volumetry of hippocampus-PC/PCC circuit: in search of early micro- and macrostructural signs of Alzheimers’s disease. Neurol. Res. Int..

[pmbaa2ad9bib070] Parkes L M, Rashid W, Chard D T, Tofts P S (2004). Normal cerebral perfusion measurements using arterial spin labeling: reproducibility, stability, and age and gender effects. Magn. Reson. Med..

[pmbaa2ad9bib071] Petersen R C, Smith G E, Waring S C, Ivnik R J, Kokmen E, Tangelos E G (1997). Aging, memory, and mild cognitive impairment. Int. Psychogeriatr..

[pmbaa2ad9bib072] Qin Y, Zhu W, Zhan C, Zhao L, Wang J, Tian Q, Wang W (2011). Investigation on positive correlation of increased brain iron deposition with cognitive impairment in Alzheimer disease by using quantitative MR R2′ mapping. J. Huazhong (Cent. China) Univ. Sci. Technol..

[pmbaa2ad9bib073] Remy F, Vayssiere N, Saint-Aubert L, Barbeau E, Pariente J (2015). White matter disruption at the prodromal stage of Alzheimer’s disease: relationships with hippocampal atrophy and episodic memory performance. Neuroimage Clin..

[pmbaa2ad9bib074] Ricci S, Fuso A, Ippoliti F, Businaro R (2012). Stress-induced cytokines and neuronal dysfunction in Alzheimer’s disease. J. Alzheimers Dis..

[pmbaa2ad9bib075] Rowley J (2013). White matter abnormalities and structural hippocampal disconnections in amnestic mild cognitive impairment and Alzheimer’s disease. PLoS One.

[pmbaa2ad9bib076] Rudko D A, Klassen L M, de Chickera S N, Gati J S, Dekaban G A, Menon R S (2014). Origins of R2^∗^ orientation dependence in gray and white matter. Proc. Natl Acad. Sci. USA.

[pmbaa2ad9bib077] Sachdev P S, Zhuang L, Braidy N, Wen W (2013). Is Alzheimer’s a disease of the white matter?. Curr. Opin. Psychiatry.

[pmbaa2ad9bib078] Salat D H (2010). White matter pathology isolates the hippocampal formation in Alzheimer’s disease. Neurobiol. Aging.

[pmbaa2ad9bib079] Santillo A F (2013). Diffusion tensor tractography versus volumetric imaging in the diagnosis of behavioral variant frontotemporal dementia. PLoS One.

[pmbaa2ad9bib080] Selnes P, Fjell A M, Gjerstad L, Bjornerud A, Wallin A, Due-Tonnessen P, Grambaite R, Stenset V, Fladby T (2012). White matter imaging changes in subjective and mild cognitive impairment. Alzheimer Dement..

[pmbaa2ad9bib081] Sen P N, Axelrod S (1999). Inhomogeneity in local magnetic field due to susceptibility contrast. J. Appl. Phys..

[pmbaa2ad9bib082] Serrano-Pozo A, Frosch M P, Masliah E, Hyman B T (2011). Neuropathological alterations in Alzheimer disease. Cold Spring Harb. Perspect. Med..

[pmbaa2ad9bib083] Sexton C E, Kalu U G, Filippini N, Mackay C E, Ebmeier K P (2011). A meta-analysis of diffusion tensor imaging in mild cognitive impairment and Alzheimer’s disease. Neurobiol. Aging.

[pmbaa2ad9bib084] Shu N, Wang Z, Qi Z, Li K, He Y (2011). Multiple diffusion indices reveals white matter degeneration in Alzheimer’s disease and mild cognitive impairment: a tract-based spatial statistics study. J. Alzheimers Dis..

[pmbaa2ad9bib085] Siemonsen S, Finsterbusch J, Matschke J, Lorenzen A, Ding X Q, Fiehler J (2008). Age-dependent normal values of T2^*^ and T2′ in brain parenchyma. AJNR Am. J. Neuroradiol..

[pmbaa2ad9bib086] Smith S M (2004). Advances in functional and structural MR image analysis and implementation as FSL. Neuroimage.

[pmbaa2ad9bib087] Smith S M (2006). Tract-based spatial statistics: voxelwise analysis of multi-subject diffusion data. Neuroimage.

[pmbaa2ad9bib088] Soares F C, de Oliveira T C, de Macedo L D, Tomas A M, Picanco-Diniz D L, Bento-Torres J, Bento-Torres N V, Picanco-Diniz C W (2014). CANTAB object recognition and language tests to detect aging cognitive decline: an exploratory comparative study. Clin. Interv. Aging.

[pmbaa2ad9bib089] Sperling R A (2011). Toward defining the preclinical stages of Alzheimer’s disease: recommendations from the National Institute on Aging-Alzheimer’s Association workgroups on diagnostic guidelines for Alzheimer’s disease. Alzheimers Dement..

[pmbaa2ad9bib090] Sudheimer K D (2014). Cortisol, cytokines, and hippocampal volume interactions in the elderly. Front. Aging Neurosci..

[pmbaa2ad9bib091] Tang X, Holland D, Dale A M, Younes L, Miller M I (2014). Shape abnormalities of subcortical and ventricular structures in mild cognitive impairment and Alzheimer’s disease: detecting, quantifying, and predicting. Hum. Brain Mapp..

[pmbaa2ad9bib092] Tuppo E E, Arias H R (2005). The role of inflammation in Alzheimer’s disease. Int. J. Biochem. Cell Biol..

[pmbaa2ad9bib093] Wakana S (2007). Reproducibility of quantitative tractography methods applied to cerebral white matter. Neuroimage.

[pmbaa2ad9bib094] Wang P N, Chou K H, Lirng J F, Lin K N, Chen W T, Lin C P (2012). Multiple diffusivities define white matter degeneration in amnestic mild cognitive impairment and Alzheimer’s disease. J. Alzheimers Dis..

[pmbaa2ad9bib095] Wang J H, Lv P Y, Wang H B, Li Z L, Li N, Sun Z Y, Zhao B H, Huang Y (2013). Diffusion tensor imaging measures of normal appearing white matter in patients who are aging, or have amnestic mild cognitive impairment, or Alzheimer’s disease. J. Clin. Neurosci..

[pmbaa2ad9bib096] Weigel M (2015). Extended phase graphs: dephasing, RF pulses, and echoes—pure and simple. J. Magn. Reson. Imaging.

[pmbaa2ad9bib097] Weiner M W (2015). 2014 Update of the Alzheimer’s disease neuroimaging initiative: a review of papers published since its inception. Alzheimers Dement..

[pmbaa2ad9bib098] Wharton S, Bowtell R (2012). Fiber orientation-dependent white matter contrast in gradient echo MRI. Proc. Natl Acad. Sci. USA.

[pmbaa2ad9bib099] Whittall K P, MacKay A L, Graeb D A, Nugent R A, Li D K, Paty D W (1997). *In vivo* measurement of T2 distributions and water contents in normal human brain. Magn. Reson. Med..

[pmbaa2ad9bib100] Whitwell J L (2012). Neuroimaging correlates of pathologically defined subtypes of Alzheimer’s disease: a case-control study. Lancet Neurol..

[pmbaa2ad9bib101] Winkler A M, Ridgway G R, Webster M A, Smith S M, Nichols T E (2014). Permutation inference for the general linear model. Neuroimage.

[pmbaa2ad9bib102] Wolk D A, Detre J A (2012). Arterial spin labeling MRI: an emerging biomarker for Alzheimer’s disease and other neurodegenerative conditions. Curr. Opin. Neurol..

[pmbaa2ad9bib103] Yablonskiy D A, Haacke E M (1994). Theory of NMR signal behavior in magnetically inhomogeneous tissues—the static dephasing regime. Magn. Reson. Med..

[pmbaa2ad9bib104] Yablonskiy D A, Reinus W R, Stark H, Haacke E M (1997). Quantitation of T-2′ anisotropic effects on magnetic resonance bone mineral density measurement. Magn. Reson. Med..

[pmbaa2ad9bib105] Zhang Y, Brady M, Smith S (2001). Segmentation of brain MR images through a hidden Markov random field model and the expectation-maximization algorithm. IEEE Trans. Med. Imaging.

[pmbaa2ad9bib106] Zhuang L, Sachdev P S, Trollor J N, Kochan N A, Reppermund S, Brodaty H, Wen W (2012). Microstructural white matter changes in cognitively normal individuals at risk of amnestic MCI. Neurology.

[pmbaa2ad9bib107] Zhuang L, Sachdev P S, Trollor J N, Reppermund S, Kochan N A, Brodaty H, Wen W (2013). Microstructural white matter changes, not hippocampal atrophy, detect early amnestic mild cognitive impairment. PLoS One.

